# Brucellosis Reactivation after 28 Years

**DOI:** 10.3201/eid1612.100678

**Published:** 2010-12

**Authors:** Önder Ögredici, Stefan Erb, Igor Langer, Paola Pilo, Anna Kerner, Horst G. Haack, Gieri Cathomas, Jürg Danuser, Georgios Pappas, Philip E. Tarr

**Affiliations:** Author affiliations: Kantonsspital Bruderholz, Basel, Switzerland (Ö. Ögredici, S. Erb, I. Langer, A. Kerner, H.G. Haack, P.E. Tarr);; University Bern, Bern, Switzerland (P. Pilo);; Cantonal Institute of Pathology, Liestal, Switzerland (G. Cathomas);; Swiss Federal Veterinary Office, Bern, (J. Danuser);; Institute of Continuing Medical Education, Ioannina, Greece (G. Pappas)

**Keywords:** Brucellosis, reactivation, chronic infection, granulomatous cholecystitis, zoonoses, bacteria, letter

**To the Editor:** Approximately 10% of patients with brucellosis experience a relapse, 90% of which occur within a year after discontinuation of antimicrobial drug therapy (*1**,**2*). Here we report a patient who had brucellosis in a disease-endemic area, immigrated to a brucellosis-free region, and experienced focal reactivation in her gallbladder 28 years later. To our knowledge, this interval is among the longest reported asymptomatic intervals between a first brucellosis episode and reactivation. The case suggests that physicians should not disregard remote histories of brucellosis and past residence in brucellosis-endemic areas when confronted with possible reactivation disease.

A woman, born in 1955, had prolonged fever without focal symptoms in 1981 and had received a diagnosis of brucellosis while living near Valencia, Spain. The brucellosis was attributed to an episode of eating unpasteurized cheese from the local dairy and was successfully treated. She immigrated to Switzerland in 1990 and was well until March 2009, when malaise and right upper quadrant pain developed, without fever. She was otherwise healthy and did not take any medication. Computed tomography (CT) scan showed a mass contiguous with the gallbladder, extending intrahepatically, with concentric calcifications and multiple gallstones (Figure). Gallbladder cancer was suspected, but when a laparotomy was performed, an acutely inflamed gallbladder with a surrounding inflammatory mass was found and excised. Gallbladder cultures on standard media (Columbia agar with 5% sheep’s blood, chocolate agar, *Brucella* blood agar, and brain-heart infusion broth) were discarded when they remained sterile after 5 days of incubation. Histopathologic examination showed granulomatous cholecystitis, and the patient was referred for infectious disease consultation. Formalin-fixed gallbladder tissue was negative for *Mycobacterium tuberculosis* complex DNA but positive for *Brucella melitensis* by PCR (*3*). Blood cultures (BACTEC Plus Aerobic/F and Anaerobic/F [Becton Dickinson, Allschwil, Switzerland], incubated for 10 days) remained sterile. No *Brucella* DNA was detected in blood and serum (*1**,**2*), and a rose bengal serum agglutination test was negative for anti-*Brucella* antibodies. Because of the rarity of the manifestation (late reactivation) and location (gallbladder), plus a residual abscess shown on CT scan 8 weeks after surgery, prolonged treatment with doxycycline and rifampin was administered for 3 months, with gentamicin added during the initial 2 weeks (*4*). Nine months after antimicrobial drug therapy was discontinued, the patient remains well.

**Figure:**
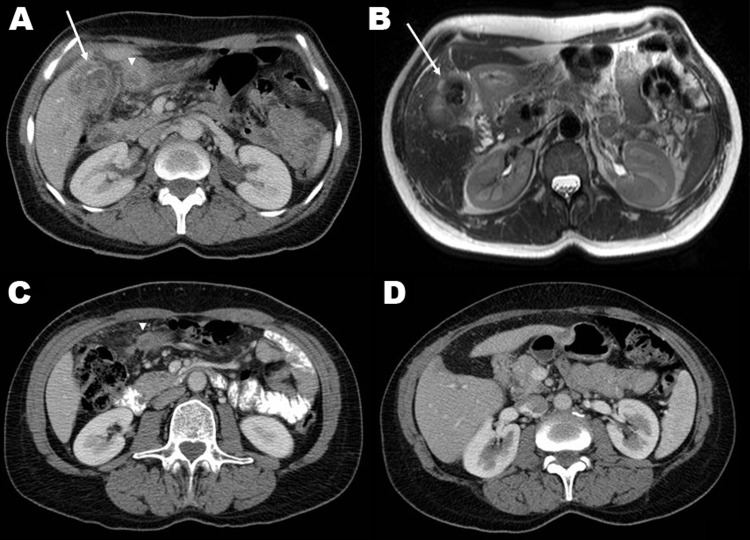
A) Contrast-enhanced computerized tomography (CT) scan showing a calcified gallbladder wall (arrow), a surrounding, calcified mass located peripherally in the liver, and an abscess in the adjacent fat tissue (arrowhead). B) T2-weighted axial magnetic resonance imaging shows multiple gallstones and a thickened gallbladder wall (arrow), inflammation and edema of the adjacent liver, fat tissue, and proximal duodenum. C) Eight weeks after cholecystectomy, contrast-enhanced CT shows a residual abscess in the adjacent fat tissue (arrowhead). D) Contrast-enhanced CT 5 months after cholecystectomy shows only minimal changes in the gallbladder bed and surrounding tissues, and no residual abscess**.**

Switzerland reported the elimination of animal brucellosis in 1963 (*5*) and has officially been brucellosis-free since 1998, according to article 14.1.2. of the Terrestrial Animal Health Code (www.oie.int/eng/normes/mcode/en_chapitre_1.14.1.htm). The last case of *B. melitensis* infection in a sheep or goat in Switzerland was reported in 1985. The annual number of human brucellosis cases reported in Switzerland remains low but seems to be increasing; 3, 5, and 13 cases were recorded in 2007, 2008, and 2009, respectively (*6*).

Most of these cases were linked to international travel or ingestion of food products imported from disease-endemic areas, e.g., Turkey. Our patient traveled annually to her family’s home town in Spain, but she vigorously denied exposure to any dairy or meat products imported from an area with known brucellosis endemicity or to any unpasteurized dairy products in general since she left Spain in 1990. Therefore, the most likely explanation was brucellosis reactivation, 28 years after the initial episode in Spain, where brucellosis was endemic. In the absence of a positive culture, brucellosis remains formally unproven. However, the granulomatous inflammation in the resected gallbladder tissue, the absence of another identified cause (e.g., tuberculosis), and the patient’s clinical and radiologic response to specific anti-*Brucella* treatment argue against the positive PCR result being merely attributable to long-term, persistent, but clinically latent, brucellosis.

Ariza (*7*) and Diaz (*8*) have reported patients with chronic hepatosplenic brucellosis after remote episodes of brucellosis (2–40 years previously). Because these patients lived in disease-endemic areas, reinfection cannot be excluded. *Brucella* organisms persist intracellularly, and their DNA has been detected in the peripheral blood of asymptomatic patients, even years after the diagnosis of clinical brucellosis and its clinically successful cure. These facts suggest that the outcome of treated brucellosis may be a chronic, persistent, asymptomatic infection, rather than complete bacterial eradication (*9*).

The pathogenesis of unusually late brucellosis reactivation in our patient remains obscure. Reactivation has only rarely been linked to underlying illnesses or immunosuppression (*2*). Rare cases of reactivation have been reported during pregnancy. *Brucella* persistence has been recorded inside calcified lesions in chronic hepatosplenic brucellosis (*7*), and adherence to foreign bodies such as joint prostheses or prosthetic heart valves and other cardiac devices may occur, perhaps in association with episodes of transient *Brucella* bacteremia (*10*). Unlike the situation with typhoid fever, no evidence links the presence of gallstones to the persistence of *Brucella* organisms in humans.

## References

[1] Pappas G, Akritidis N, Bosilkovski M, Tsianos E. Brucellosis. N Engl J Med. 2005;352:2325–36. 10.1056/NEJMra050570 15930423

[2] Ariza J, Corredoira J, Pallares R, Viladrich PF, Rufi G, Pujol M, Characteristics of and risk factors for relapse of brucellosis in humans. Clin Infect Dis. 1995;20:1241–9. 10.1093/clinids/20.5.1241 7620005

[3] Hinić V, Brodard I, Thomann A, Cvetnić Z, Makaya PV, Frey J, Novel identification and differentiation of *Brucella melitensis, B. abortus, B. suis, B. ovis, B. canis*, and *B. neotomae* suitable for both conventional and real-time PCR systems. J Microbiol Methods. 2008;75:375–8. 10.1016/j.mimet.2008.07.002 18675856

[4] Ariza J, Bosilkovski M, Cascio A, Colmenero JD, Corbel MJ, Falagas ME, ; International Society of Chemotherapy. Institute of Continuing Medical Education of Ioannina. Perspectives for the treatment of brucellosis in the 21st century: the Ioannina recommendations. PLoS Med. 2007;4:e317. 10.1371/journal.pmed.0040317 18162038 PMC2222927

[5] Corbel MJ. Brucellosis: an overview. Emerg Infect Dis. 1997;3:213–21. 10.3201/eid0302.970219 9204307 PMC2627605

[6] Swiss Federal Veterinary Office. Swiss zoonoses report 2008 [cited 2010 Oct 9]. http://www.bvet.admin.ch/dokumentation/00327/02466/02762/index.html?lang=en

[7] Ariza J, Pigrau C, Canas C, Marãn A, Martãnez F, Almirante B, Current understanding and management of chronic hepatosplenic suppurative brucellosis. Clin Infect Dis. 2001;32:1024–33. 10.1086/319608 11264030

[8] Diaz R, Ariza J, Alberola I, Casanova A, Rubio MF. Secondary serological response of patients with chronic hepatosplenic suppurative brucellosis. Clin Vaccine Immunol. 2006;13:1190–6. 10.1128/CVI.00086-06 16943345 PMC1656543

[9] Navarro E, Segura JC, Castano MJ, Solera J. Use of real-time quantitative polymerase chain reaction to monitor the evolution of *Brucella melitensis* DNA load during therapy and post-therapy follow-up in patients with brucellosis. Clin Infect Dis. 2006;42:1266–73. 10.1086/503035 16586386

[10] Weil Y, Mattan Y, Liebergall M, Rahav G. *Brucella* prosthetic joint infection: a report of 3 cases and a review of the literature. Clin Infect Dis. 2003;36:e81–6. 10.1086/368084 12652405

